# Lifestyle and environmental factors may induce airway and systemic inflammation in firefighters

**DOI:** 10.1007/s11356-022-22479-x

**Published:** 2022-09-12

**Authors:** Joanna Orysiak, Magdalena Młynarczyk, Robert Piec, Agnieszka Jakubiak

**Affiliations:** 1 Central Institute for Labour Protection – National Research Institute, Czerniakowska St. 16, 00-701 Warsaw, Poland; 2 Institute of Internal Security, The Main School of Fire Service, Słowackiego St. 52/54, 01-629 Warsaw, Poland; 3 Department of Heart Failure and Cardiac Rehabilitation, Medical University of Warsaw, Żwirki and Wigury St. 61, 02-091 Warsaw, Poland

**Keywords:** Air pollution, Firefighters, Immune response, Respiratory infection, Smoke exposure

## Abstract

Health status depends on multiple genetic and non-genetic factors. Nonheritable factors (such as lifestyle and environmental factors) have stronger impact on immune responses than genetic factors. Firefighters work is associated with exposure to air pollution and heat stress, as well as: extreme physical effort, mental stress, or a changed circadian rhythm, among others. All these factors can contribute to both, short-term and long-term impairment of the physical and mental health of firefighters. Increased levels of some inflammatory markers, such as pro-inflammatory cytokines or C-reactive protein (CRP) have been observed in firefighters, which can lead to local, acute inflammation that promotes a systemic inflammatory response. It is worth emphasizing that inflammation is one of the main hallmarks of cancer and also plays a key role in the development of cardiovascular and respiratory diseases. This article presents possible causes of the development of an inflammatory reaction in firefighters, with particular emphasis on airway inflammation caused by smoke exposure.

## Introduction

The basic tasks of the State Fire Service include identifying, organizing, and conducting rescue operations during fires in rural areas and forests, as well as in an urban environment (e.g., residential or industrial buildings). Those tasks also include: liquidation of local threats or natural disasters (Act of August 24, 1991 on the State Fire Service; US Department of Labor 2015; Barros et al. 2021).

Factors affecting the safety of firefighters under fire conditions include not only high temperature and radiation heat flux density, smoke, oxygen deficiency, or damage to the structure of the buildings or its components, but also harmful combustion products (Sawicki 2004). Compounds released during the combustion of various types of materials (including biomass, plastic-based materials and textiles and fuels) (Valavanidis et al. 2008; Navarro et al. 2019; Altshuler et al. 2020; Jaffe et al. 2020; Barros et al. 2021) have properties that are toxic, corrosive, flammable, explosive or allergenic to the human body (Krzemińska and Szewczyńska 2020). Moreover, due to variable combustion conditions, the combination of released different compounds, e.g., wood smoke and diesel fuel, may act additively or even synergistically in its overall toxic effect (Adetona et al. 2017). It is worth noting that some of the compounds released during fire are classified as known, probable or possible carcinogens (Fabian et al. 2014; IARC 2021; Barros et al. 2021). Outdoor air pollution is recognized as one of the major contributors to human disease, as most people live in urban areas where air pollution far exceeds the values recommended by the World Health Organization (WHOa n.d.; Turner et al. 2020). Earlier studies showed that with increasing air pollution caused by fires, the number of doctor’s visits increases due to problems with the respiratory and cardiovascular systems (Moore et al. 2006; Orozco-Levi et al. 2006; Kurmi et al. 2010; Smith et al. 2011; Hejl et al. 2013; Reid et al. 2016). Moreover, outdoor air pollution, especially with particulate matter (PM), contributes to the increase in lung cancer incidence and mortality (Turner et al. 2020; Arias-Pérez et al. 2020). In Poland, the air is one of the most polluted in the European Union (WHOa n.d.; Gładka and Zatoński 2016), which causes premature deaths (over 47,000 a year) and shortens life on average by a year among people of southern Poland (Maas and Grennfelt 2016; Gładka and Zatoński 2016). Although the International Agency for Research on Cancer (IARC) has identified outdoor air pollution as carcinogenic to humans, a recent IARC report has classified firefighting as only a possible carcinogen (Group 2B) (IARC 2013, 2021; Barros et al. 2021). However, some studies (Daniels et al. 2014; Jalilian et al. 2019; Soteriades et al. 2019; Pinkerton et al. 2020; Laroche and L'Espérance 2021; Smith et al. 2021) indicated a significant increase in the incidence of, among others, rectal, prostate, bladder and testicular cancer, as well as mesothelioma and malignant melanoma in firefighters compared to the general population. Cancer as well as cardiovascular disease (sudden cardiac death (SCD) causes about 45% of on-duty fatalities) are the leading cause of death in this occupational group (International Association Of Fire Fighters—IAFF n.d.; Soteriades et al. 2011; Daniels et al. 2014; Smith et al. 2019b).

Firefighters work is associated with exposure to air pollution and heat stress, as well as with, among others: extreme physical effort (intensive physical exercise), mental stress, or a changed circadian rhythm (Santo de Oliveira et al. 2012; Angelo and Chambel 2015; Soteriades et al. 2011, 2019; Kim et al. 2020; Barros et al. 2021). All the above factors can contribute to both, short-term and long-term impairment of the physical and mental health of firefighters (Barros et al. 2021) (Table 1).

**Table 1:** Short-term and long-term health consequences of the work of a firefighter (Guidotti et al. 2011; Soteriades et al. 2011; Daniels et al. 2014; Smith et al. 2019b; Cohen et al. 2019; Brackbill et al. 2019; Groot et al. 2019; Jalilian et al. 2019; Soteriades et al. 2019; Pinkerton et al. 2020; Laroche and L’Espérance 2021; Smith et al. 2021; Barros et al. 2021; Cleven et al. 2021)

Short-term health problems	Long-term health problems
Decreased lung function	Development of cardiovascular diseases, e.g., - Myocardial infarction - Stroke - Unstable angina
Decreased vascular function	Development of respiratory diseases, e.g., - Asthma - Obstructive pulmonary disease
Arteriosclerosis	Cancer, e.g., - Thyroid cancer - Lung cancer - Leukemia
Altered cardiovascular parameters	Mental disorders, e.g., - Post-traumatic stress disorder (PTSD)
Inflammation	
Development of allergic airway sensitization	

It was observed that diseases such as cancer, lung disease, heart disease, and post-traumatic stress disorder appear to be more common in World Trade Center terrorist rescue workers than in the general population, even now over 20 years later (Smith et al. 2021), and the risk of cancer death (e.g., in lung cancer and leukemia) increases with longer and frequent firefighters’ exposure to the fire environment (Daniels et al. 2015). The impact of occupational exposure of firefighters on their health has been intensively studied in recent years, but still the evidence suggesting a cause-and-effect relationship is limited and unclear (Barros et al. 2021). Therefore, the aim of this study was to describe selected causes of the development of an inflammatory reaction in firefighters, with particular emphasis on the effect of exposure to smoke on inflammation in the respiratory tract. However, it should be emphasized that not only firefighters may develop inflammation related to the specificity of their work. Workers of other uniformed services, as well as representatives of other professions (e.g., from the construction industry), also work in unfavorable conditions (long working hours, stress, shift work, extreme environmental conditions, air pollution) (Ulvestad et al. 2001; Ramey et al. 2012; Violanti et al. 2017).

## Airway and systemic inflammation in firefighters

It is well known that health status depends on multiple genetic and nongenetic factors (Morales et al. 2022). It was shown that nonheritable factors (such as lifestyle and environmental factors) have stronger impact on immune responses than genetic factors (Brodin et al. 2015; Morales et al. 2022). The exposure to nongenetic factors on individual-level (physical activity, body mass and composition, diet, psychological stress, sleep and circadian rhythms, smoking, gut microbiome) and general level (climate and sunlight, environmental pollution) collectively is the so-called exposome (Niedzwiecki et al. 2019; Morales et al. 2022). These exposures could act synergistically through common mechanisms, which could impact the inflammatory response (Furman et al. 2019; Morales et al. 2022).

The inflammatory reaction (inflammation; inflammatory response) is a normal, physiological response of the body to the invasion of a pathogen or the appearance of a factor damaging tissues or organs (Całkosiński et al. 2009; Kuzior and Gorczyca 2010). Inflammatory reaction may appear at both local (usually) and systemic level (Całkosiński et al. 2009). The factors that could cause inflammation include infectious and noninfectious factors such as (Całkosiński et al. 2009; Chen et al. 2017; Bennett et al. 2018):

- Physical factors (e.g., mechanical, ionizing radiation, magnetic field, ultrasound waves, burns, frostbite, physical injury, foreign bodies, severe life-threatening injuries (trauma))
- Chemical agents (e.g., turpentine, carrageenan, acids, bases, glucose, fatty acids, toxins, alcohol, chemical irritants (including fluorine, nickel, and other trace elements))
- Biological agents (e.g., bacteria, viruses, fungi, protozoa, exotoxins, endotoxins, damaged cells)
- Psychological factors

The above factors, acting for a sufficiently long time on the body’s tissues, disturb their local homeostasis, which triggers inflammation aimed at neutralizing the harmful factor, repairing damage, accelerating wound healing and restoring the previous state (Ahmed 2011; Całkosiński et al. 2009; Kuzior and Gorczyca 2010).

The first local clinical symptoms—swelling (tumor), redness (rubor), pain (dolor), increased temperature (calor), impaired function (functio laesa), as well as diagnostic indicators (inflammatory markers) appear in a relatively short time (minutes) from the onset of inflammation (Całoksiński et al. 2009; Maśliński and Gajewski 2014; Chen et al. 2017) (Table 2).

**Table 2:** Examples of pro- and anti-inflammatory markers according to Chen et al. (2017) and Bennett et al. (2018)

Pro-inflammatory markers	Anti-inflammatory markers
IL-1α (interleukin 1 alfa)	IL-4 (interleukin 4)
IL-1β (interleukin 1 beta)	IL-10 (interleukin 10)
TNF-α (tumor necrosis factor alfa)	IL-11 (Interleukin 11)
IFN-γ (interferon gamma)	TGF-β (transforming growth factor beta)
IL-6 (interleukin 6)	Cortisol
IL-8 (interleukin 8)	Lipoxin
IL-12 (interleukin 12)	
IL-17 (interleukin 17)	
GM-CSF (granulocyte–macrophage colony-stimulating factor)	
HSP-70 (heat shock proteins 70)	
CRP (C-reactive protein)	
Coagulation factors	
Prostaglandins	
Leukotrienes	

**Fig. 1:**
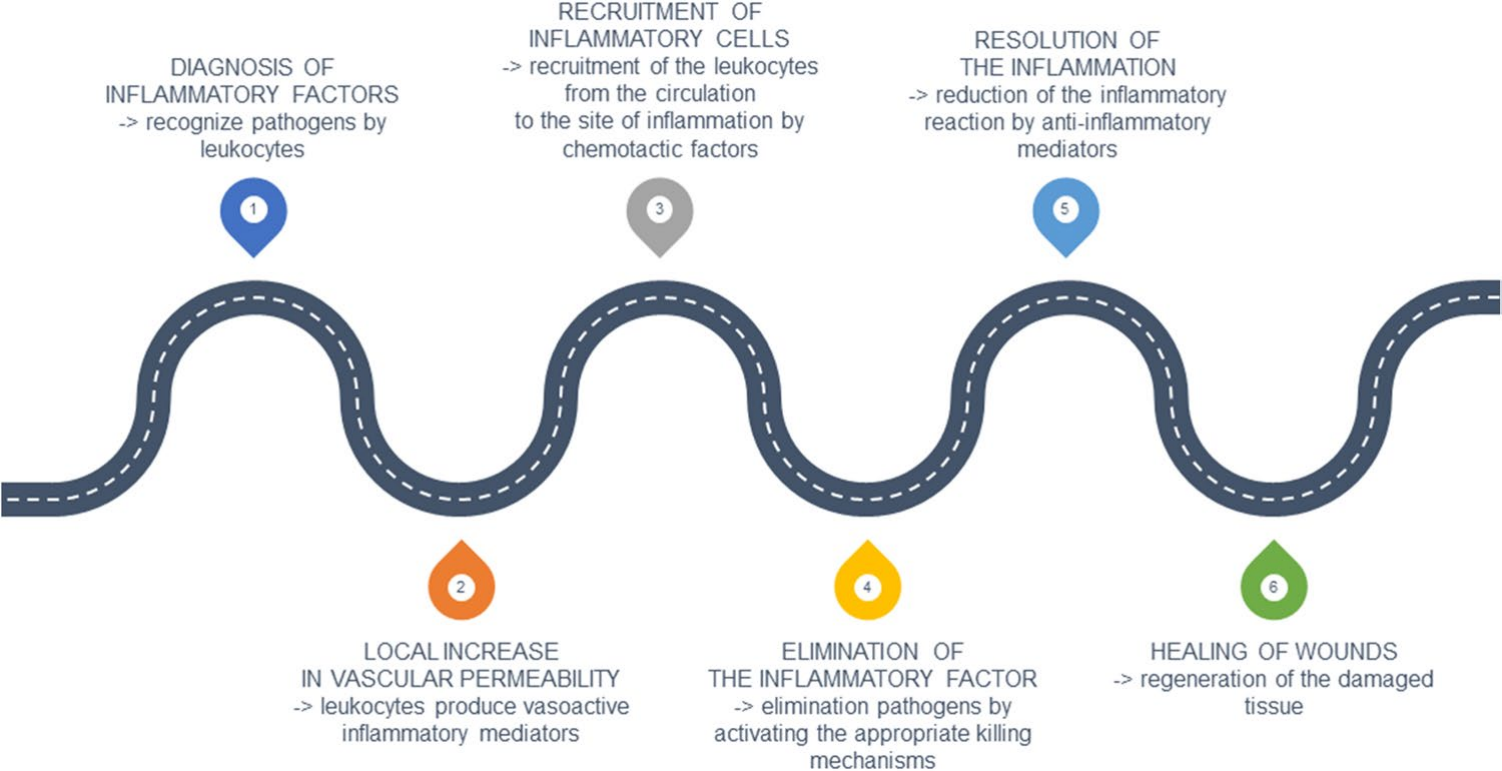
The scheme of the processes of the inflammatory reaction in the body (according to Ahmed 2011; Całkosiński et al. 2009; Kuzior and Gorczyca 2010; Chen et al. 2017; Kołaczkowska 2007)

Although the processes of the inflammatory reaction depend on the initial stimulus and its location in the body, they all have a common mechanism (shown in Fig. 1).

The mechanism is characterized by the following stages (Ahmed 2011; Całkosiński et al. 2009; Kuzior and Gorczyca 2010; Chen et al. 2017; Kołaczkowska 2007):

1. Diagnosis of inflammatory factors—leukocytes in the tissue recognize pathogens entering the body by pathogen-associated molecular patterns (PAMPs). PAMPs are recognized by the so-called pattern recognition receptors (PRRs), which are found, for example, on the surface of phagocytic cells. Some PRRs can also recognize endogenous signals that are activated during tissue or cell damage (DAMPs, danger-associated molecular patterns). DAMP and PAMP share receptors such as toll-like receptor 4 (TLR4), which may suggest a similarity between infectious and non-infectious inflammatory responses.
2. Local increase in vascular permeability—leukocytes in the tissue begin to produce vasoactive inflammatory mediators.
3. Recruitment of inflammatory cells by chemotactic factors released by leukocytes in tissue—recruitment of leukocytes from the circulation to the site of inflammation using proinflammatory cytokines (TNF-α, IL-1β and IL-6) and chemokines (e.g., IL-8/CXCL8, MCP-1/CCL2, MIP-1α/CCL3) as well as other chemoattractants. Leukocytes involved in the inflammatory reaction by chemotaxis, marginalization, rolling, adhesion, and diapedesis (leukocyte passing beyond the blood vessel, extravasation) enter the site of injury from the bloodstream. The first cells attracted to the site of inflammation are neutrophils (key mediators of the inflammatory response; their increase is noted at the 4th hour of the inflammatory response), followed by monocytes/macrophages, lymphocytes (NK cells, T cells, and B cells) and mast cells.
4. Elimination of the inflammatory factor—leukocytes at the site of inflammation eliminate pathogens by activating the appropriate killing mechanisms (aerobic and anaerobic).
5. Resolution of the inflammation—anti-inflammatory mediators (e.g., anti-inflammatory cytokines, soluble receptors for pro-inflammatory cytokines, components of the coagulation and fibrinolytic system, as well as glucocorticoids) reduce the inflammatory reaction and leukocytes are degraded. However, if the inflammatory factor is not removed, chronic inflammation can develop.
6. Healing of wounds—after elimination of the inflammatory factor, in the case of tissue damage, the healing process begins, for instance, by the stimulation of fibroblasts and the formation of a network of collagen fibers filling the defect caused by inflammation. However, new tissue possesses up to a maximum of 80% of the tissue strength before damage.

Moderate inflammation is a beneficial reaction for the body due to the inhibition of bleeding caused by trauma, removal of necrotic products, excretion of endotoxins and exotoxins, as well as the creation of a demarcation line that limits inflammation (Całkosiński et al. 2009). However, uncontrolled acute inflammation could turn into chronic inflammation, thus contributing to the development of many chronic diseases, including cardiovascular and digestive diseases, diabetes, arthritis, and cancer (Chen et al. 2017; Marchewka et al. 2018) (Table 3).

**Table 3:** Characteristics of acute and chronic inflammation (Maśliński and Gajewski 2014; Pahwa et al. 2021)

Characteristic	Acute inflammation	Chronic inflammation
Duration	Brief, usually from a few minutes to several days	Months or even years
The cause of inflammation	Most often as a result of infection	• Often as a result of infection (failure to eliminate the causative agent of acute inflammation) • Exposure to low levels of a specific irritant or foreign material, such as industrial chemicals • Autoimmune diseases
Cells in the inflammatory infiltrate	Neutrophils predominate	Mainly lymphocytes, macrophages and plasma cells

It is worth emphasizing that inflammation is one of the main hallmarks of cancer and also plays a key role in the development of cardiovascular and respiratory diseases (Singh et al. 2019; Greten and Grivennikov 2019; Barros et al. 2021). Increased levels of some inflammatory markers were observed in firefighters, e.g., pro-inflammatory cytokines or C-reactive protein (CRP), which may lead to occurrence local, acute inflammation that promotes a systemic inflammatory response (Gianniou et al. 2018; Barros et al. 2021), and thus may develop of cancer or other diseases (Han et al. 2018) (Table 4).

**Table 4:** Examples of inflammatory markers that may have potential impact on the development of cancer, cardiovascular diseases, or respiratory diseases in firefighters (Eagan et al. 2010; Nolan et al. 2012; Gaughan et al. 2014; Cho et al. 2014; Kwon et al. 2016; Chen et al. 2017; Singh et al. 2018; Bennett et al. 2018; Sproston and Ashworth 2018; Zeig-Owens et al. 2018; Smith et al. 2019a; Han et al. 2020; Barros et al. 2021; Kong et al. 2021; Broman et al. 2021; Cleven et al.^2021^; Weiden et al. 2021)

Cancer	Cardiovascular diseases	Respiratory diseases
VCAM-1 (vascular cell adhesion molecule)	Leukocytes and their populations, e.g., monocytes, neutrophils, eosinophils	Interleukins, e.g., IL-4, IL-5, IL-6, IL-8, IL-10, IL-13, IL-21
WBC (White blood cells; leukocytes)	Interleukins, e.g., IL-1β; IL-6, IL-10, IL-18	Leukocytes and their populations, e.g., macrophages, neutrophils, lymphocytes T, eosinophils
CRP (C-reactive protein)	ICAM-1 (intercellular adhesion molecule-1)	TNF-α (tumor necrosis factor-alpha)
SAA (serum amyloid A)	VCAM-1 (vascular cell adhesion molecule 1)	CRP/hs-CRP (C-reactive protein/high sensitivity C-reactive protein)
	hs-CRP (high sensitivity C-reactive protein)	sTNFR-1 (soluble tumor necrosis factor receptor 1)
	TNF α and β (tumor necrosis factor-alpha and beta)	GM-CSF (granulocyte–macrophage colony-stimulating factor)
	P-Selectin	MDC (macrophage-derived chemokine)
	MMP-9 (matrix metalloproteinase-9)	
	TAC (total antioxidant capacity)	

**Fig. 2:**
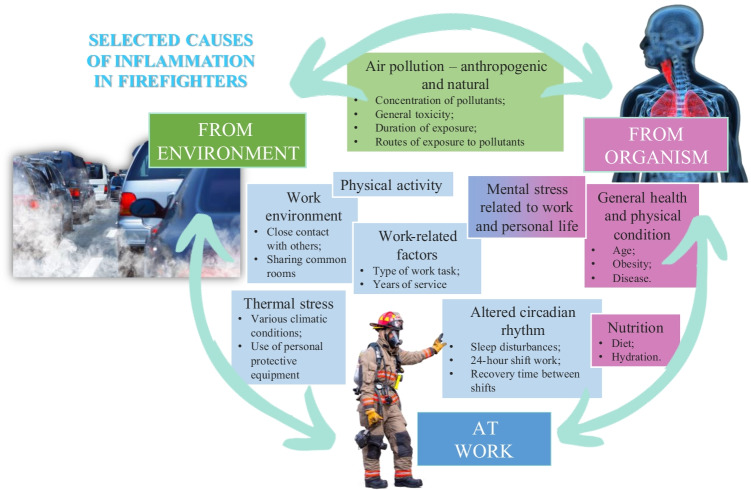
Selected causes of inflammation in firefighters (Wolkow et al. 2015; Adetona et al. 2016; Billings and Focht 2016; Adetona et al. 2017; Watkins and Richardson 2017; Keaney et al. 2018; Walsh 2018; Watkins et al. 2019; Dolsen et al. 2019; Jang et al. 2020; Barros et al. 2021; Savall et al. 2021) (own source, used photos from SciePro/Bigstockphoto and Canva)

The selected reasons for the development of inflammation in firefighters are shown in Fig. 2.

In recent years, more and more studies have been conducted on the development of inflammation and its health effects during firefighting activities, in volunteers not related to the fire service (Ferguson et al. 2016; Morris et al. 2020) and among firefighters (Swiston et al. 2008; Greven et al. 2012; Hejl et al. 2013; Gianniou et al. 2016, 2018; Adetona et al. 2017) or fire instructors (Watt et al. 2016; Watkins et al. 2019). They included real-life studies during normal work (Swiston et al. 2008; Greven et al. 2012; Hejl et al. 2013; Gianniou et al. 2016, 2018; Adetona et al. 2017) or training courses to become a firefighter (Andersen et al. 2018a) and in laboratory conditions, e.g., in smoke/heat chambers (Smith et al. 2005; Walker et al. 2015a,b; Ferguson et al. 2016; Watkins et al. 2019; Morris et al. 2020). In these studies, various pro and anti-inflammatory markers in blood or other biological material (e.g., urine, sputum) were determined (Barros et al. 2021). Although several studies showed the development of inflammation to fire exposure, the mechanisms of this immune response are still being investigated (Adetona et al. 2017).

### Air pollution—anthropogenic and natural pollutants

The increase in human exposure to various pollutants—xenobiotics, including air pollutants, hazardous waste products, and industrial chemicals—as well as the increase in climate change-related fires is driven by the rapid development of urbanization (Furman et al. 2019; Prunicki et al. 2019). Xenobiotics are chemical substances that are not naturally produced or expected to occur in human body, but there are present in it (Morales et al. 2022). They have negative impact on immune cells and may cause inflammation (Pollard et al. 2018; Morales et al. 2022). One of the xenobiotics is tobacco smoking and high rates of smokeless tobacco (SLT) use have been observed among firefighters (Phan et al. 2022).

Air pollution is a mixture of anthropogenic (industrial emissions, air/road/ship traffic, residential heating, and construction) and natural pollutants (Woodby et al. 2021; Bachmann et al. 2020). In urban environment, air pollution consists of, among others, nitrogen oxides (NOx), sulfur oxides (SOx), tropospheric ozone (O_3_), volatile organic compounds (VOCs), particulate matter (PM), CO (carbon oxide), and lead (Pb) (Woodby et al. 2021; Bachmann et al. 2020). In turn, fire smoke is a complex mixture containing hundreds of compounds, e.g. carbon monoxide, particulate matter (PM 2.5), nitrogen dioxide, sulfur dioxide, ozone, acrolein, formaldehyde, benzene, toluene, xylene, hydrogen cyanide, and hydrogen chloride (Adetona et al. 2016; Krzemińska and Szewczyńska 2020). The composition of the smoke from the fire differs depending on the combustion conditions (Adetona et al. 2016) (Table 5).

**Table 5:** Selected compounds and their concentration present in test fire environment (Pośniak and Skowroń 2000; BAZA CHEMPYŁ n.d.; Sawicki 2004; Krzemińska and Szewczyńska 2020)

Name of the chemical (CAS No.)	Observed concentration (mg/m^3^)	The highest permissible chemical concentrations (mg/m^3^)			Toxicological information
		NDS-MAC (TWA): maximum allowable concentration	NDS-MAC (STEL): maximum allowable short-term concentration	NDS-MAC (C): maximum allowable ceiling concentration	
Carbon monoxide CO [630–08-0]	to 31,320	23	117	-	Toxic and other harmful biological effects on the human body: toxic gas, suffocating, binds to hemoglobin of blood cells and inhibits tissue respiration
Carbon dioxide CO_2_ [124–38-9]	18,000–110,000	9000	27,000	**-**	Toxic and other harmful biological effects on the human body: physically suffocating gas (by reducing oxygen partial pressure)
Hydrogen cyanide HCN [74–90-8]	0–80	1	-	5	Toxic and other harmful biological effects on the human system: very toxic, suffocating substance—blocks tissue respiration; irritating
Hydrogen chloride HCl [7647–01-0]	to 300	5	10	**-**	Toxic and other harmful biological effects on the human body: toxic and corrosive substance. Accustomed workers tolerate HCI concentration of 15 mg/m^3^
Benzene C_6_H_6_ [71–43-2]	3850	1.6	-	-	Toxic and other harmful biological effects on the human system: toxic substance, damages the hematopoietic system, carcinogenic; has a narcotic and local irritating effect
Formaldehyde HCHO [50–00-0]	19	0.37	0.74	-	Toxic and other harmful biological effects on the human body: toxic or harmful substance, irritating and sensitizing

Abbreviations: NDS-MAC (TWA): maximum allowable concentrations–the time-weighted average concentration for a conventional 8-h workday and 42-h workweek (Pośniak and Skowroń 2000); NDS-MAC (STEL): maximum allowable short-term concentrations–the short-term exposure limit being an average concentration for an exposure, which may last together no longer than 30 min during a workday (Pośniak and Skowroń 2000); NDS-MAC (C): maximum allowable ceiling concentrations–concentration, which should not be exceeded even momentarily (Pośniak and Skowroń 2000)

One of the most studied pollutants in terms of health effects is PM (particulate matter)—a mixture of liquid, solid, or solid and liquid particles suspended in the air (Woodby et al. 2021). Depending on the size, PM is divided into PM10 (thoracic; diameter less than 10 microns), PM2.5 (respirable; diameter less than 2.5 microns) or ultrafine particles (UFP) with PM diameter of less than 0.1 microns (generated in combustion and biogenic processes) (Woodby et al. 2021). It seems that smaller PM (e.g., PM2.5 and UFP included diesel exhaust particles) could have more negative/harmful effects on health, because they penetrate deeper into the lungs and adsorb more chemicals to their surface. It was shown that at cellular and systemic levels the onset of inflammation and oxidative stress is caused by UFP (Woodby et al. 2021). It follows that, toxicity induced by pollutants is mainly caused by increasing oxidative stress, which results in increased inflammation (main pathophysiological mechanism induced by air pollution) (Woodby et al. 2021; Bachmann et al. 2020). Pollution toxic agents may also activate the aryl hydrocarbon receptor (AhR), chronic activation of which may lead to immunotoxicity (Bachmann et al. 2020; Suzuki et al. 2020).

The World Health Organization (WHO) described that 9 of 10 people in the world breathe polluted air exceeding the WHO recommended values for ambient air quality (WHOb n.d.; Hahad et al. 2021). Consequently, air pollution (including fire smoke particles) is one of the major contributors to disease and increased morbimortality, especially cancer, cardiovascular disease and respiratory disease (Bachmann et al. 2020; Hahad et al. 2021). It was observed that wildfire particulate matter may impact respiratory health more than PM from other sources (Aguilera et al. 2021). Inhalation of smoke particles may trigger an inflammatory reaction in the respiratory system. This inflammation may be associated with the development of respiratory tract infections (e.g., bronchitis or pneumonia), and that may contribute to the development of lung cancer (IARC 2010; Andersen et al. 2018b; Navarro et al. 2019, 2021). Exposure to smoke may contribute to the development of viral diseases as it leads, among others, to the induction of oxidative stress and inflammation as well as airway hyperresponsiveness, which disturb the homeostasis of the respiratory system and thus make it more susceptible to infections (Loaiza-Ceballos et al. 2021). Nowadays, due to the ongoing COVID-19 pandemic, firefighters are also especially exposed to the development of this disease, and the number of deaths due to COVID-19 includes a significant number of firefighters (Fire rescue 1 n.d.). Firefighters are not only on the front lines of the fight against SARS-CoV-2, which means that they are directly exposed to the disease, but also the specificity of their work (e.g. exposure to smoke) may contribute to an additional, often multiplied risk of SARS-CoV-2, as well as the severity of COVID-19 disease in this occupational group (Navarro et al. 2021; Graham et al. 2021).

In the following review, the authors focused on the studies on an inflammatory response caused by exposure to smoke in firefighters under real conditions during their work, and some of these studies are discussed below.

#### Methods

This review is focused on the scientific literature published in the last 15 years (2005–2021). PUBMED database was searched using the following keywords: firefighters, systemic inflammation, inflammation, local inflammation, fire exposure, immune system, wood smoke, smoke exposure. Initially, regardless of the year, we received 174 publications. After the elimination of duplicates (*n* = 90) and analyzing abstracts, the following exclusion criteria were applied:

1. Publications up to 2005 (excluding 2005)
2. Animal research
3. Nonoriginal research/review study
4. Not related to airway or systemic inflammation
5. Exposure during firefighting training and activities simulating occupational work (tests performed in laboratory conditions, e.g., smoke/heat chamber not during “normal” operation)
6. Not related to firefighters
7. Research considering the impact of supplementation on immune response
8. Noninclusion of biological samples

A total of 10 studies were selected.

#### Results

The selected articles are listed in Table 6.

**Table 6:** Analysis of studies examining the impact of exposure to fire on respiratory and systemic inflammation in firefighters

Study	Participants	Study design—blood sample collection	Immune markers in blood	Pre- to post-shift significant changes in blood immune markers—impact of fire exposure	Other examination in blood	Immune markers in sputum or other biological samples	Pre- to post-shift significant changes in immune markers—impact of fire exposure	Other examination
Adetona et al. 2017	*N* = 12 (10 firefighters and 2 volunteers; 9 male and 3 female)	Pre- to post-work shift on prescribed burns and non-burn day	White blood cells and their populations, IL-1β, IL-6, IL-8, TNF-α, CRP, SAA, ICAM-1, VCAM-1	↑ IL-8 concentration, SAA concentration and CRP concentration depend on work tasks	Work task impacted the cross-shift changes in immune markers	Not studied	Not studied	Not studied
Andersen et al. 2018b	*N* = 22 male professional firefighters	Before and after a 24-h works shift	SAA, CRP, ICAM-1, VCAM-1, IL-6, IL-8	↑ VCAM-1 concentration	IL-6 and IL-8 were generally below the detection limit	Not studied	Not studied	Not studied
Gianniou et al. 2016	*N* = 92 male firefighters (63 full-time professional firefighters, 29 trainees that had been working on a part-time basis for at most one year and 18 healthy subjects—control group)	Examination of maximum occupational exposure of 1 year compared to the long-term exposure	IL-8, IL-4, IL-13, VEGF, TNF-α, ECP	Not studied	↑ IL-8 and TNF- α levels in professional firefighters as compared to the trainees and control group, as well as, in trainees compared to healthy subjects Correlation between the years in service and VEGF levels in serum The impact of duration of the occupation in service and inflammation	Total number of cells and percentage of: eosinophils, neutrophils, macrophages and lymphocytes in sputum and BALF IL-4, IL-8, IL-13, VEGF, TNF-α and ECP in sputum	Not studied	↑Eosinophils in professional firefighters compared to trainees’ and control group in sputum ↑ Eosinophils in professional firefighters compared to trainees’ in BAL ↑ IL-8, ECP, VEGF and TNF- α levels in sputum of professional firefighters as compared to the trainees and control group ↑ IL-8 and TNF- α concentrations in trainees than in healthy subjects The impact of duration of the occupation in service and inflammation – longer duration in service higher number of cells in sputum and BAL, higher percentage of eosinophils, neutrophils and lymphocytes in sputum Correlation between the years in service and ECP and VEGF levels in sputum
Gianniou et al. 2018	*N* = 60 forest firefighters	After 24–48 h post-exposure (after forest firefighting operation for several days) and 3 months after exposure (off-season)	IL-8, IL-4, IL-13, TNF- α, VEGF, ECP	Not studied	↑ IL-8, VEGF and TNF- α levels post-exposure in comparison to off-season More intense systemic inflammation in firefighters who participated in fire operation continuously > 10 h (38 ± 22 h) than < 10 h (6 ± 3 h)	Total number of cells and percentage of: eosinophils, neutrophils, macrophages and lymphocytes in sputum and BALF IL-4, IL-8, IL-13, VEGF, TNF-α and ECP in sputum	Not studied	↑ Neutrophils and eosinophils, as well as, IL-8 and TNF- α levels in sputum post-exposure in comparison to off-season ↑ Neutrophils in BAL post-exposure in comparison to off-season
Greven et al. 2012	*N* = 51 firefighters (43 male, 8 female)	Within 24 h following exposure to fire smoke, and after a week and 3 months	CC16, SPA, IL-1β, IL-6, IL-8, IL-10, TNF-α, INF-γ	↑ IL-8 concentration	↑ IL-8 concentration 1 week post-exposure and 3 months post-exposure	Sputum Total cell count, Percentage of: eosinophils, lymphocytes, macrophages, neutrophils, basophils and bronchial epithelial cells, particle count, between 1 and 5 days following exposure	↑ neutrophils	Not studied
Hejl et al. 2013	*N* = 12 wildland firefighters (10 male and 2 female)	Pre- to post-work shift on prescribed burns and pre-shift on non-burn day	IL-1β, IL-6, IL-8, TNF-α, CRP, SAA, ICAM-1, VCAM-1	↑ IL-8 concentration ↑ IL-8, CRP and ICAM-1 in > 50% of samples across work shift	Work task impacted the cross-shift changes in immune markers	Not studied	Not studied	Not studied
Main et al. 2020	*N* = 38 male volunteer firefighters	Before and after 12-h firefighting shift	IL-1β, IL-2, IL-6, IL-8, IL12P70, GM-CSF, TNF-α, INF-γ, IL-4, IL-5, IL-7, IL-10, IL-13	↑ IL-6, IL-8 concentrations ↓ IL-10 concentration	Not studied	Not studied	Not studied	Not studied
Swiston et al. 2008	*N* = 52 seasonal forest firefighters (49 male; 3 female)	Baseline samples in training camps before firefighters were dispatched to fire zones; exposure samples—after a day of fire-fighting; exercise samples—after a day of physical activity without fire exposure	White blood cells and their populations, band cells, IL-6, IL-8, GM-CSF, MCP-1, CRP	↑ Total white blood cell counts, PMN and band cell counts ↑ IL-6 and IL-8 concentrations ↑ MCP-1 concentration	↑ White blood cell and PMN counts after strenuous exercise without fire-fighting	Sputum granulocytes, monocytes, lymphocytes, macrophages or bronchial epithelial cells. Macrophages were subdivided into those with no visible inclusions and those with less than, or greater than, 5% of their cytoplasm containing dust particles, labelled as negative, low-positive or high-positive macrophages	↑ Granulocytes (mostly neutrophils) ↑ Inclusion-positive macrophages ↓ Inclusion-negative macrophages ↑ Low positive-inclusion macrophages	Not studied
Watkins et al. 2021a	*N* = 53 fire service instructors (47 male, 6 female) *N* = 57 operational firefighters (55 male, 2 female)	Examination the baseline immune markers between fire service instructors and operational firefighters—before any heat exposure that day of sample collection and a minimum of 12 h after the last fire exposure	White blood cells and their populations, platelet count, IL-6, TNF-α, IL-1β, CRP, IgG, cTnT	Not studied	↑ Neutrophil counts, platelet count, basophil counts, the neutrophil-to-lymphocyte ratio, cTnT, IL-6, IL-1β, CRP and IgG concentrations in fire service instructors compared to operational firefighters ↑ Eosinophil count in operational firefighters than in fire service instructors The more frequent exposure to fire, the greater the increase in cytokine levels	Not studied	Not studied	Not studied
Wu et al. 2020	*N* = 12 (9 males, 3 females)	Not studied	Not studied	Not studied	Not studied	Exhaled breath condensate (EBC) were collected before (pre-shift), after (post-shift) and the morning following the prescribed burn shifts (burn days) as well as regular work shifts (non-burn days) IL-6, IL-8, CRP, sICAM-1, 8-isoprostane	↑ 8-Isoprostane (*p* = 0.06)	No significant change

Abbreviations: ↑, increase/more; ↓, decrease/less; BAL, bronchoalveolar lavage; BALF, bronchoalveolar lavage fluid; CC16, Clara cell protein; CRP, C-reactive protein; cTnT, cardiac troponin T; EBC, Exhaled breath condensate; ECP, eosinophil cationic protein; GM-CSF, ganulocyte-macrophage colony-stimulating factor; ICAM-1, intercellular adhesion molecule 1; IgG, immunoglobulin G; IL, interleukin; INF-γ, interferon gamma; MCP-1, monocyte chemotactic protein 1; PMN, neutrophils; SAA, serum amyloid A; sICAM-1, soluble intercellular adhesion molecule 1; SP-A, surfactant protein A; TNF-α, tumor necrosis factor-alpha; VCAM-1, vascular cell adhesion molecule 1; VEGF, vascular endothelial growth factor

**Fig. 3:**
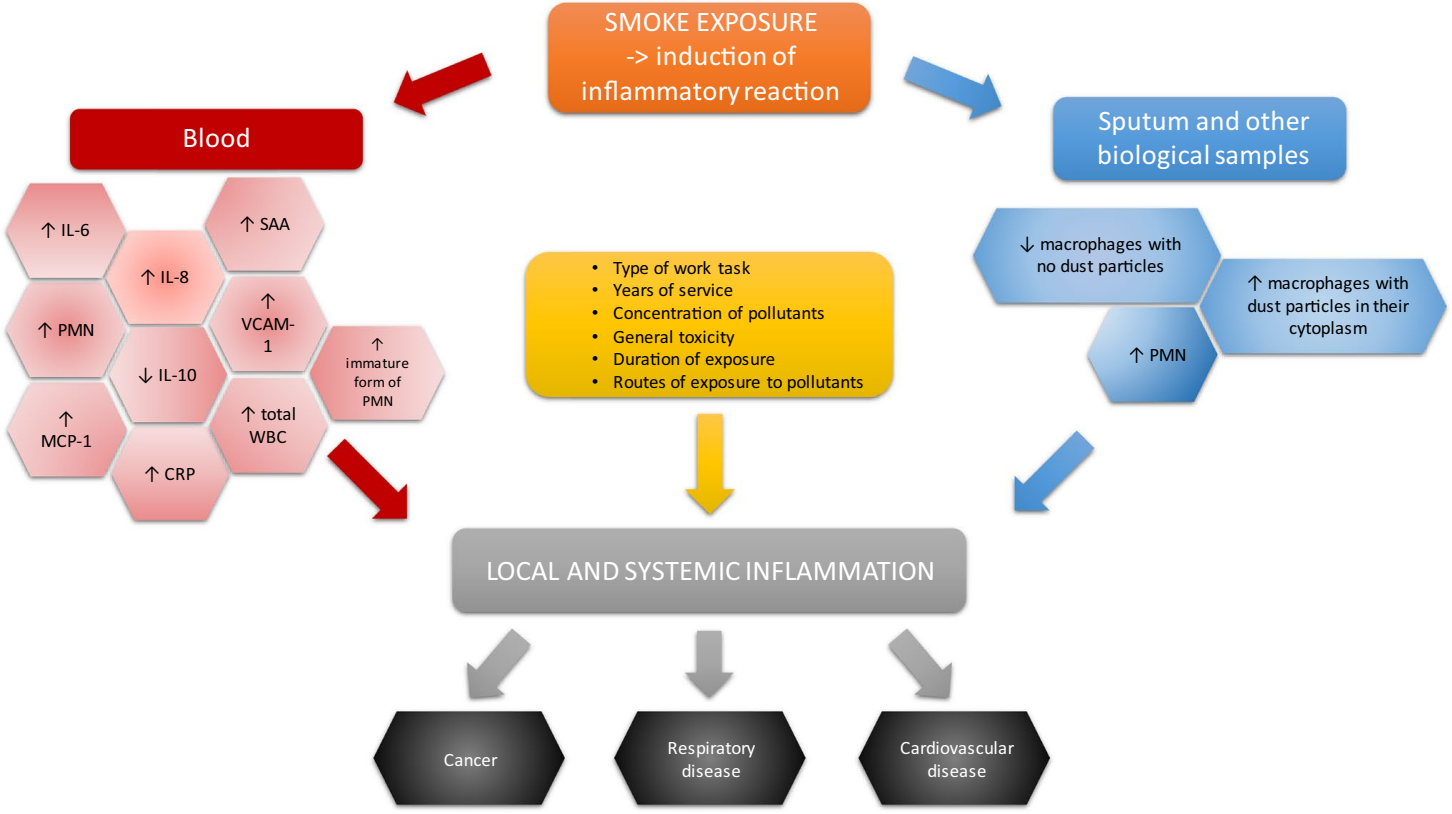
The impact of smoke exposure on the development of inflammatory reaction in firefighters (Adetona et al. 2017; Andersen et al. 2018b; Greven et al. 2012; Hejl et al. 2013; Main et al. 2020; Swiston et al. 2008)

The analysis of the articles (Table 6; Fig. 3) allowed for the selection of several main factors that may affect the human immune system. The influence of the above-mentioned factors on the inflammatory response in firefighters is discussed below.

#### Respiratory symptoms

Symptoms of both systemic and local inflammation in the respiratory tract (increased percentage of neutrophils in the blood and the influx of neutrophils in the bronchial and bronchoalveolar lavage) were observed in young healthy people (not related to the fire department), who were exposed to wood smoke particles (Ghio et al. 2012). Similarly, acute exposure to forest fire smoke causes the development of lung/respiratory inflammation (mainly neutrophilic in nature) as well as a systemic inflammatory response in 52 firefighters (including 3 women) (Swiston et al. 2008). In this study, biological samples were collected during training camps (baseline tests), after a day of firefighting (exposure), or after a day of physical activity without exposure to smoke (exercise) (Swiston et al. 2008). Respiratory symptoms (the most common: coughing, sputum production, and nasal congestion) were observed in 65% of firefighters as a result of smoke exposure (Swiston et al. 2008). It is worth noting that Swiston et al. (2008) observed relatively mild respiratory symptoms. The clinical significance of these symptoms may be of minor relevance in a healthy population; however, in at-risk individuals (e.g., those with asthma or chronic obstructive pulmonary disease), such airway irritation may worsen the lung condition (Swiston et al. 2008). Moreover, during those tests, the concentration of IL-6, IL-8, and MCP-1 in blood increased after the ending of the firefighting operation. Also, in the case of leukocytes, the percentage of sputum granulocytes (mainly neutrophils) increased significantly after firefighting shift, as well as the total number of leukocytes, neutrophils (PMN) and young, immature forms of neutrophils in the blood. However, the increase in white blood cell counts, including neutrophils in the blood, was also caused by intensive physical activity without exposure to smoke in firefighters. Therefore, it was suggested that the increase in the number of these cells may be due to a rather non-specific response to physical stress / exercise rather than exposure to smoke (Swiston et al. 2008). However, the relationship between air pollution and white blood cell counts, and hence its possible impact on the systemic inflammatory response, was also confirmed in a study examined of over 10,000 Taiwanese people (Hung et al. 2021). On the other hand, an increase in the concentration of IL-6, IL-8, and the count of immature neutrophils was observed only after smoke exposure, suggesting the development of an exposure-induced systemic inflammation (Swiston et al. 2008). Increased the number of circulating immature neutrophils in the blood in firefighters suggested the bone marrow stimulation for enhanced release of granulocytes (Tan et al. 2000; Swiston et al. 2008), which is also confirmed by other studies among people exposed to smoke haze during forest fires (Tan et al. 2000).

Airway inflammation, mainly related to an increase in the percentage of sputum neutrophils, was also observed in firefighters within 5 days after exposure to fire smoke (Greven et al. 2012). This study also demonstrated the development of long-term systemic inflammation, which was manifested by an increase in IL-8 concentration 24 h after smoke exposure and lasted up to 3 months after exposure (Greven et al. 2012). Similarly, in a study of 60 firefighters participated in forest fighting operations continuously for several days, a higher incidence of respiratory symptoms (such as wheezing, cough, chest tightness, sneezing, and expectoration) and pulmonary function impairment were observed after firefighting compared to the off-season (the interval between post-exposure and off-season measurements was 3 months) (Gianniou et al. 2018). The above observations were related to the development of systemic inflammation. Higher levels of IL-8 and TNF-α, both in sputum and blood, were showed in firefighters after fire exposure compared to the off-season period. There were also changes in the percentage of the leukocyte populations—percentage of neutrophils and eosinophils were higher after smoke exposure than off the fire season (Gianniou et al. 2018).

#### Type of work tasks

Inflammation was also observed in a study of 12 firefighters (10 men and 2 women) of the American Forest Service from the Savannah River Site in South Carolina during four prescribed burns (Hejl et al. 2013). Blood samples were collected before and after work during the 4 “fire” days and before work on 1 day without fires (non-burn day). This study showed that the concentrations of IL-8, CRP and ICAM-1 increased in over 50% of the subjects after work shift compared to before work shift. Moreover, it was observed that changes in the immune system (changes in IL-8 concentration) were related to the work tasks. Firefighters who were involved in “lighting” (involves the ignition of the forest understory) showed the highest increase in IL-8 concentration before and after work shift compared to firefighters responsible for “holding” (involves maintaining the fire within pre-established boundaries) or “lighting/other” (Hejl et al. 2013).

The type of work task has an impact on response of the immune system also during the examination of 12 firefighters—10 working for the United States Forest Service Savannah River and 2 volunteers entitled to work with prescribed burns (9 men, 3 women) (Adetona et al. 2017). The biological samples were collected from people working on prescribed burns and on days without fires. The tasks performed by firefighters were divided into 4 groups: “holding” working in defense/preventing the spread of fire, “lighting” actively extinguishing the fire and “non-burn day–exposures” and “non-burn day–office.” It was shown, inter alia, that despite the lack of differences in the concentration of particulate matter (PM) 2.5 exposure, firefighters lighting had 36% more inhaled total PM2.5 than firefighters responsible for the holding. In addition, lighters showed an over 1.3 times greater increase in IL-8 concentration after than before work, compared to holders. Similarly, in firefighters lighting there was a 1.3 times greater increase in SAA concentration, as well as 1.1 times greater increase in CRP concentration compared to those who were holding before and after the work shift.

#### Time/years of exposure

Short-time exposure to various size fractions of particulate matter is sufficient to induce the systemic inflammatory reaction and subclinical decrement in distal lung function in general population (Tsai et al. 2012; Hassanvand et al. 2017; Dauchet et al. 2018). Moreover, among the general population longer exposure to high levels of air pollution (elevated PM10 values) was associated with increased inflammation (Tsai et al. 2019).

Similarly for firefighters, the degree of inflammation is related to exposure/working time, both from direct exposure and years in service. Gianniou et al. (2018) showed that continuously participation in forest firefighting action > 10 h (38 ± 22 h [mean ± standard deviation]) caused increased systemic inflammation in firefighters compared to exposure < 10 h (6 ± 3 h) (Gianniou et al. 2018).

The impact of lower exposure and shorter work shifts was also observed in the study of Adetona et al. (2017). Differences in the immune response were observed depending on the work tasks performed by firefighters in Adetona et al. (2017) and Hejl et al. (2013) studies. However, the weaker immune system response (smaller differences in IL-8 levels before and after work) in study from 2017 may be due to lower exposure and shorter work shifts. Personal exposures to PM2.5 and CO among firefighters was 2.5 and 4.3 times higher in the Hejl et al. (2013) study than Adetona et al. (2017). Active prescribed burning time was shorter in the study of 2017 than 2013 (averaged 4.5 h as opposed to 6.2 h).

Another study also found that number of years in service was related to the degree of inflammation in firefighters. As many as 92 firefighters (63 of them were full-time professional firefighters, and 29 were training firefighters and worked part-time for a maximum of 1 year) and 18 healthy people (control group) participated in the study (Gianniou et al. 2016). There was no difference between professional firefighters and trainees in the use of respiratory protective equipment as well as in work actions performed by them. In this study, a higher incidence of general respiratory symptoms (coughing, dyspnea, sneezing) was observed in professional firefighters compared to trainees. In addition, the group of professional firefighters had a higher percentage of eosinophils in sputum and BAL (bronchoalveolar lavage) compared to trainees. The sputum of professional firefighters was also found to have higher levels of IL-8, ECP, VEGF, and TNF-α than in trainees and control subjects, and serum concentration of IL-8 and TNF-α was also higher compared to trainees. It is worth noting that the trainees had significantly higher levels of IL-8 and TNF-α in sputum and serum than the control group. This study also showed that longer duration of the occupation in service (exposure years) was associated with higher sputum eosinophils, neutrophils, and lymphocytes (Gianniou et al. 2016). It may be associated with the development of allergic bronchial sensitization, chronic airway inflammation, as well as systemic inflammation and the presence of chronic respiratory symptoms (Gianniou et al. 2016).

It is noteworthy that firefighters are at risk of developing both local and systemic airway inflammation at an early stage in their working lives. Even a year’s exposure in the group of trainees to firefighters resulted in a measurable increase in the rates of local and systemic inflammation markers compared to healthy subjects (Gianniou et al. 2016). In addition, it was shown that the inflammatory reaction in the respiratory tract and systemic inflammation increase with years of service. This is confirmed not only by studies of inflammatory markers (e.g., in sputum and serum) but also by bronchial biopsy, in which inflammation of the lower respiratory tract was documented. It was shown that the more years of service are associated with the more neutrophilic infiltrates and changes in the bronchial epithelium (Gianniou et al. 2016).

Another group that is regularly exposed to fire scenarios, and at the same time not directly related to firefighting, are fire service instructors (Watkins et al. 2021a). Fire service instructors, who are responsible for conducting training reported an average of around 13 fire exposures per month, compared to active firefighters with an average of 1 fire over the same period (Watkins et al. 2018). Another study reported a maximum of 8 per week and 20 per month fire exposures among fire instructors, while among active firefighters none was involved in more than 3 exposures per month (Watkins et al. 2021a). For this reason, fire instructors may also develop inflammation due to occupational exposure. It was observed that the instructors had increased levels of neutrophils, platelets, basophils, and a higher neutrophil-to-lymphocyte ratio (NLR) as well as higher levels of cardiac troponin T (cTnT), IL-6, IL-1β, CRP and IgG compared to firefighters. This study also found over 7 times greater risk of various diseases in instructors than in firefighters (Watkins et al. 2021a). Elevated rates of inflammation in instructors suggested the presence of chronic systemic inflammation, which may favor the development of e.g. cardiovascular diseases (Watkins et al. 2021a). In the case of the disease caused by SARS CoV-2, the levels of e.g. IL-6, CRP, platelets, neutrophils, and NLR were significantly increased in patients with a very severe form of COVID-19 (Shang et al. 2020; Sayah et al. 2021; Wang et al. 2020). However, due to the lack of research, it is not possible to conclude whether high levels of these inflammatory markers in instructors may affect the form of COVID-19.

Among Fire Service Instructors, it was also observed that more frequent exposure to fire, similar to professional firefighters, promotes the development of more severe inflammation (higher levels of proinflammatory cytokines) (Watkins et al. 2021a). Fire instructors who participated in more than 9 fire scenarios a month were more likely to have elevated levels of IL-6, IL-1β, CRP, and IgG than those with lower fire exposure. In addition, instructors are also more than 15 times more likely to develop symptoms such as fatigue, sleep disturbances, headaches, colds and flu-like symptoms (Watkins et al. 2021a).

### Specific type of work

Firefighter’s immune system is not only impacted by direct exposure to fire. Altered circadian rhythm caused by sleep disturbances, 24-h shift work or inadequate regeneration between shifts had an impact on disorders in the proper functioning of the body.

Adequate sleep, which for adults amounts to 7–9 h of quality sleep per night, is needed for optimal functioning (Hirshkowitz et al. 2015; Frost et al. 2021), optimal physical performance, cognitive function or well-being as well as reduction of the risk of musculoskeletal injury or illness (Frost et al. 2021; Robinson et al. 2021). Additionally, improper sleep quality and quantity may impair productivity at work and increase the risk of accidents (Chattu et al. 2018; Romero Cabrera et al. 2021).

Nowadays, poor sleep hygiene may be caused by insufficient hours of sleep, long working hours, shift work, frequent awakenings, disordered breathing, difficulty falling asleep, low sleep efficiency, feelings of day-time sleepiness, sleep medication, and artificial light (Frost et al. 2021; Mollayeva et al. 2016; Morales et al. 2022). It was shown that sleep quality and well-being may be negatively affected by prolonged periods of shift working (Turner et al. 2018). Decrease in resting metabolic rate and altered blood glucose concentrations (Buxton et al. 2012; Turner et al. 2018), as well as fatigue-induced reductions in physical activity in shift workers may lead to being overweight and obese (Turner et al. 2018; Romero Cabrera et al. 2021). Night-shift workers were also exposed to artificial light, especially the blue spectrum. This exposure after sundown may cause disturbance in circadian rhythm and thus may promote inflammation (Furman et al. 2019; Morales et al. 2022), because exposure to light is associated with reduced melatonin synthesis—an important immunomodulator with anti-inflammatory properties (Mauriz et al. 2013; Morales et al. 2022).

Circadian rhythm and sleep play an important role in regulating homeostasis of the immune system by influencing both innate and adaptive immune responses (Haspel et al. 2020; Morales et al. 2022). A meta-analysis showed, that sleep disturbance and the extreme of long sleep duration (> 8 h per night) were associated with higher levels of CRP and IL-6 (Irwin et al. 2016). However, study into the relationship between sleep disturbance and markers of inflammation is inconclusive, which may be due to different methods of assessing sleep parameters (Dolsen et al. 2019).

Firefighters are the group in which poor sleep quality and sleep disturbances are highly frequent (Lim et al. 2014; Barger et al. 2015; Billings and Focht 2016; Frost et al. 2021; Savall et al. 2021). Sleep disorders in firefighters are associated with among others musculoskeletal symptoms, shift work, depression, frequency of emergency and off-duty work (Lim et al. 2014; Billings and Focht 2016; Jang et al. 2020). It was shown that the 24on/48off shift schedule was associated with the best sleep quality (Billings and Focht 2016). On the other hand, working at second job impairs/negatively affects the sleep quality in firefighters (Billings and Focht 2016). In addition, married firefighters often chose off-duty life and family/home obligations rather than focusing on reducing sleep debt (Watkins et al. 2021b). Moreover, it was observed, that firefighters with short sleep duration (≤ 6 h per night) and bad sleepers were heavier, had higher BMI and increased body fat in comparison to participants with normal sleep duration or good sleepers, which leads to higher prevalence of hypertension and obesity in this group (Romero Cabrera et al. 2021).

### Mental stress

Firefighters are exposed not only to physical stress (related to the work tasks) but also to mental stress.

Psychological stress may cause immune dysfunction (Glaser and Kiecolt-Glaser 2005; Morales et al. 2022). The effect of stress on immune function depends on the duration of the stress. Acute stressors (lasting minutes) may upregulate natural immunity, but decrease specific immunity (Segerstrom and Miller 2004). Short-term stress tends to attenuate cellular immunity, but not humoral immunity, whereas long-term stress (chronic stress) may suppress both cellular and humoral immunity (Segerstrom and Miller 2004; Morales et al. 2022). The increase of some inflammatory markers (e.g., IL-1β, IL-6, IL-8, IL-10, TNF-α, CRP, cortisol) was associated with acute or chronic stress (Marsland et al. 2017; Noushad et al. 2021; Morales et al. 2022). It was observed that psychological stressors may cause physiologic changes, leading to systemic chronic inflammation and poor health due to impaired ability of glucocorticosteroids to effectively reduce inflammation (lower sensitivity due to high continuous elevation of cortisol) (Cohen et al. 2012; Furman et al. 2019; Bachmann et al. 2020). Moreover, psychological stress may also increase the risk of respiratory infection or other disease (Takkouche et al. 2001; Morales et al. 2022), promote “inflammaging” (Yegorov et al.^2020^; Morales et al. 2022) as well as decrease immunity during prolonged heavy exercise and heavy training (Walsh 2018).

Firefighters’ work stressors may involve performing risky work task frequently in unstable, unfamiliar work zones with infectious/chemical hazards as well as long-lasting (24 h) shifts with rapid reaction periods between rest and emergency response (MacDermid et al. 2021). Due to the specific nature of the work of firefighters, a high level of stress during workdays was noted (Santo de Oliveira et al. 2012) and the severity of experienced stress increased with years of service (Santo de Oliveira et al. 2012; Makara-Studzińska et al. 2020). Firefighters are exposed to high rates of traumatic or stressful events during service. More than half of the Canadian career firefighters participating in the study declared that they were exposed to death in the line of duty in the past year, including death of a child (14%) (Nazari et al. 2020). Rodrigues et al. (2018) observed, that although 'fire' situations were more common during shifts, 'accidents' were more stressful for firefighters. Firefighters suffer from mental disorders, such as post-traumatic stress disorder or depression (Santo de Oliveira et al. 2012; Harvey et al. 2016). Their incidence is even higher in retired firefighters (8% and 5% for current firefighters, 18% and 18% for retired firefighters, respectively) and increases with the number of fatal incidents (trauma exposure) (Santo de Oliveira et al. 2012; Harvey et al. 2016). Moreover, cardiorespiratory fitness, arterial stiffness and sleep quality were correlated with firefighters’ occupational stress, which is an important factor behind the elevated risk of cardiovascular diseases in this occupational group (Yook 2019).

### Thermal stress (operation in various climatic conditions—cold and hot environments; use of PPE)

Thermal stress in firefighters may be caused by hard work in unfavorable microclimate conditions or indirectly caused by the use of personal protective equipment (PPE) in the above-mentioned conditions. Studies conducted among athletes did not confirm a greater threat to the immune system when exercising in heat conditions compared to exercise in thermoneutral conditions. In addition, people who exercise in heat tend to feel fatigue more quickly, so their exposure to heat stress tends to be self-limiting (Gonzalez-Alonso et al. 1999; Walsh et al. 2011). However, it should be remembered that physical exercise usually lasts up to about 2 h, and work usually takes much longer, therefore the disturbance in the immune system due to heat in workers may be greater than in athletes.

In the study of firefighters, an increase in core temperature (+ 1.4 ± 0.5 °C) was associated with an increase in leukocytes, neutrophils, platelets, and TNFα levels after heat testing in heat chamber (air dry-bulb temperature 100 ± 5 °C), during two 20-min simulated search tasks and while wearing structural firefighting personal protective clothing (PPC) and self-contained breathing apparatus (SCBA) (Walker et al. 2015a). Some of these markers remained elevated up to 24 h (Walker et al. 2015a,b).

The body’s heat load is influenced not only by the high external temperature, but also by the severity of the work performed, as well as the specific clothing used by firefighters. Tests of thermal parameters of special clothing for firefighters showed that clothing provides a barrier to heat exchange between the user and the surrounding environment (Młynarczyk and Zielińska 2020; Młynarczyk 2020). The special clothing for firefighters has a multilayer construction (Nayak et al. 2014; Młynarczyk 2020), consists of: an external fabric layer; a membrane; a thermal insulation layer; and lining. It should be noted that the layers protect against: flame, heat, chemicals and mechanical injuries, against water and other liquids, like chemicals, blood-borne pathogens and also against environmental heat. On the one hand, a high value of thermal insulation is desirable, but on the other, the use of highly insulating clothing impairs the exchange between heat produced by a person and the surrounding environment (Nayak et al. 2014). It should also be noted that personal protective equipment, including clothing, is quite heavy (e.g., the combined weight of PPC and SCBA was c.a. 22.0 kg (Walker et al. 2015a) and constitutes an additional burden on the body. Marszałek and Młynarczyk (2021) observed that even a short-lasting, 30-min effort in air temperature of 30 °C (in climatic chamber doing activities close to those undertaken during rescue operations) while wearing special clothing for firefighters can result in high levels of physiological reactions (Marszałek and Młynarczyk 2021). Dorman and Havenith (2009) analyzed the impact of the weight and structure of protective clothing. According to these authors, the metabolic cost of the expended physical effort may increase from 2.4% even to 20.9%, when different types of protective clothing are used in comparison to the test conditions.

It is also important for firefighters that heat exposure may worsen cardiovascular and respiratory diseases. There were two possible mechanisms by which heat exacerbates respiratory disease (Anenberg et al. 2020). First, systemic and pulmonary inflammation increase by heat as a consequence of thermoregulation. Secondly, heat may impair breathing patterns need to compensate for elevations in body temperature (White 2006; Anderson et al. 2013; Anenberg et al. 2020). It should also be noted that an increase in skin temperature by every 5 °C may increase the absorption of harmful compounds by 400% (Stec et al. 2020).

### General health and physical condition (e.g., age, obesity, disease)

#### General health—chronic infections and diseases

Certain diseases, such as diabetes, are also associated with the development of inflammation. Insulin resistance causes various immune system responses that exacerbate inflammation and may lead to hyperglycemia. Disturbances in both the innate and acquired immune responses may attenuate the functioning of the immune system and promote the development of infectious diseases in people with diabetes (Berbudi et al. 2020). On the other hand, lifelong infections caused by cytomegalovirus, Epstein–Barr virus, hepatitis C virus, and other infectious agents may contribute to systemic chronic inflammation; however, its impact on immune dysregulation remains controversial (Furman et al. 2019).

#### Age

During age chronic, sterile, low-grade inflammation (“inflammaging”) was developed (Franceschi et al. 2018). It was also observed that immunosenescence—an age-related functional decline of the immune system manifested, among others, by a decreased response to novel antigens, results in higher susceptibility to infectious diseases (Bennett et al. 2018). Upregulation of inflammatory markers with age may also cause functional decline of multiple organs and may contribute to the pathogenesis of age-related diseases (Navarro et al. 2016; Bennett et al. 2018; Franceschi et al. 2018).

#### Sex/gender

It is known that the functioning of the immune system may differ between men and women, which may translate into epidemiological differences in the occurrence of various diseases, e.g., asthma, autoimmune diseases, or cancer (Bachmann et al. 2020; Dolsen et al. 2019). Some markers of inflammation (e.g., CRP) have been shown to be higher in women, although the data were inconclusive (Navarro et al. 2016). It has been suggested that changes in inflammatory activity and responses between sexes may be at least in part due to hormonal differences (Dolsen et al. 2019).

#### Body mass index/body mass

According to WHO (World Health Organization) (WHOc n.d.; Morales et al. 2022), 39% of adults in the world are overweight and 13% are obese. Obesity has negative effects on health, among other because it is associated with accelerated aging and subsequent dysfunction of the immune system (Morales et al. 2022).

The effect of obesity on immune function may result from increased level of proinflammatory cytokines and other molecules released by adiposity, which may lead to low-grade chronic inflammation and its chronic consequences (Makki et al. 2013; Navarro et al. 2016; Morales et al. 2022). Moreover, leptin (most abundant adipokines) may impact both innate and adaptive immunity (Maurya et al. 2018; Morales et al. 2022) by increasing the proinflammatory cytokine production as well as CRP (Graßmann et al. 2017; Morales et al. 2022). In obesity, the leptin resistance (increase level of leptin) is often observed, which is associated with disruption in production of cytokine, increased susceptibility to infectious and autoimmune diseases, and upregulated inflammatory responses (Maurya et al. 2018; Morales et al. 2022). It should also be noted that the expression of angiotensin-converting enzyme (ACE) 2, important entry receptor for SARS-CoV-2, is greater in adipose tissue than in lung tissue, which in the case of excess adipose tissue may contribute to promote viral entrance into target cells and increased the risk of COVID-19 infection (Morales et al. 2022). Moreover, excess adipose tissue may be associated with respiratory dysfunction (impair ventilatory mechanics) leading to the decrease of both functional residual capacity and total lung capacity (Grassi et al. 2020; Morales et al. 2022). It was also shown that proinflammatory state tends to be exacerbated in stress outcomes in obese individuals (Bennett et al. 2018).

The prevalence of obesity and overweight was alarmingly high among firefighters (Munir et al. 2012; Barry et al. 2020; Chizewski et al. 2021). In American firefighters, according to the World Health Organization BMI (Body Mass Index) categorization, only 13.1% of firefighters were of normal weight, 51.2% were overweight, and 35.7% were obese (Durand et al. 2011). In Poland, 5% of firefighters were obese, 42% were overweight and 53% had normal weight (Nowak et al. 2018). In turn, 55.1% of male UK firefighters were overweight, 16.3% were obese and 28.6% had normal weight (Turner et al. 2018). Studies of obese workers (not only in the fire department) showed an increased rate of diseases and injuries, workers’ compensation claims and absenteeism rate, lower productivity at work (work performance), higher work limitations, early retirement, and increased mortality (Kuehl et al. 2012).

### Work environment (close contact with others, sharing common rooms—increased risk of infection)

People-to-people close contact at work in enclosed spaces with limited amount of breathing air promotes the spread of pathogens, which may increase the risk of developing various infections, and thus initiate/enhance inflammation. Moreover, illness risk, and hence, inflammatory response, may increase when firefighters share bottles or equipment, without proper hygiene, which inhibits pathogen transmission (Keaney et al. 2018; Moriyama et al. 2020).

### Physical activity

Physical inactivity is a major problem among different populations nowadays (Furman et al. 2019; Morales et al. 2022). It has been shown that as many as 1/4 of the population worldwide does not meet the minimum international requirements for physical activity (i.e., at least 150 min/week of moderate or 75 min/week of vigorous aerobic activities) (Guthold et al. 2018; Morales et al. 2022), especially in high-income countries (Furman et al. 2019). Physical inactivity is associated with the increase of inflammatory markers, such as IL-1β, IL-6, IL-7, TNF and C-reactive protein (Filgueira et al. 2021). The lack of physical activity negatively affects not only inflammation but also insulin resistance, lipid metabolism, cardiovascular function, and muscle failure (sarcopenia) (Morales et al. 2022).

On the other hand, the positive effect of moderate regular physical activity on immune function is visible in reduction of community-acquired infectious and non-infectious diseases due to anti-inflammatory effects of exercise (Gleeson et al. 2011; Chastin et al. 2021; Morales et al. 2022). Conversely, a decline in both innate and acquired immunity is observed after intense exercise during the recovery period (Walsh 2018). There was some evidence, that the risk of infectious diseases increased with prolonged and intensive exercise, while moderate activity reduced this risk compared with a completely sedentary state (Gleeson et al. 2011; Nieman and Wentz 2019). A high level of cardiorespiratory fitness (CRF), resulting from regular physical exercise, is associated with fewer days of illness from acute respiratory infection and less expression of immune markers that may reduce the risk of respiratory illness complications (Zbinden-Foncea et al. 2020; Morales et al. 2022).

Firefighting is a very physically demanding occupation. According to the classification of the US Department of Labor (2015), the physical demands in firefighting were determined as “very hard”—the highest of five levels of physical demands. Moreover, the energy requirements for a specific firefighting task are also high, which means high intensity of physical activity (metabolic equivalent of task (MET)—for “victim rescue” and “automobile accident” was 6.8 MET and for “fire suppression” was 8.0 MET) (Ainsworth et al. 2011; Jang et al. 2020). Firefighting requires high levels of physical fitness (Stevenson et al. 2017) and the importance of better physical fitness (greater aerobic capacity and muscular endurance) for the effective performance of fire-fighting tasks is emphasized (Chizewski et al. 2021). Inadequate levels of physical fitness in this occupational group may increase the health risk to firefighters and the public (Stevenson et al. 2017). Maintaining good physical condition by engaging in regular physical activity is essential in firefighters’ work to maintain their aerobic capacity, endurance and muscular strength necessary for the safe and effective firefighting (Stevenson et al. 2017; Turner et al. 2018). However, research into regular physical activity in this group is inconclusive. Some authors emphasized the high physical activity of firefighters, meeting the minimum requirements at least 150 min of physical activity per week according to the American College of Sports Medicine’s (ACSM) physical activity guidelines (Garber et al. 2011), others observed that most firefighters are not active enough (Durand et al. 2011; Turner et al. 2018; Barry et al. 2020). Selected health (body fat percent) and fitness parameters (push-up capacity, pull-ups, and running time) of the recruits were shown to improve during training at the fire academy. However, these effects were not maintained after graduation in probationary firefighters as a result of decrease of physical activity and increased TV screen time. However, it should be noted that blood pressure increased throughout the study period (Lan et al. 2021). For this reason, some authors indicated the need for the implementation of physical activity and nutrition outreach programs, which would increase physical activity in this group and reduce the incidence of overweight and obesity (Martin et al. 2019; Barry et al. 2020; Gendron et al. 2020).

It should be noted that regular exercise at least 4 to 5 times a week may prevent excessive decline of aerobic capacity related to work demands in firefighters (Punakallio et al. 2012). Moreover, physical exercise and high levels of cardiorespiratory fitness may be associated with lower risk of cardiovascular disease (Baur et al. 2011; Strauss et al. 2021) as well as may have a positive impact on coping with psychological stress related to work in firefighting (Throne et al. 2000).

The effect of physical activity on the inflammatory response in firefighters can be two-way. On the one hand, low physical activity is noted in a large percentage of firefighters, which may contribute to the development of obesity and inflammation. On the other hand, intense physical exertion that firefighters perform while on duty with too short recovery between intense efforts (Ronsen et al. 2002; Smith 2003) may contribute to the disruption of the immune system and increase the risk of various diseases in this group.

### Incorrectly balanced diet, including the lack of adequate hydration of the body

In recent years, increased consumption of processed or ultra-processed food with significant amounts of salt, fat, sugar or flavor additives has been observed (Furman et al. 2019; Morales et al. 2022). Changes in eating habits towards the Western diet may lead to inflammation by increasing proinflammatory and oxidative stress markers as well as may adversely change the microbiome (Furman et al. 2019; Morales et al. 2022). On the other hand, Western eating habits tend to show a low consumption of vegetables and fruit. This has a negative effect on health as fruit and vegetables contain microbiota-accessible carbohydrates (low intake of their consumption may result in a decrease in the richness and diversity of microbiota), bioactive compounds as well as minerals and vitamins that are involved in preventing or attenuating oxidative and inflammatory stress and improve the overall immune function (Iddir et al. 2020; Gombart et al. 2020; Morales et al. 2022). Immune function can be influenced by minerals and vitamins, such as among others zinc, copper, iron, and selenium, vitamins A, B6, B12, and D (Gombart et al. 2020; Morales et al. 2022). The role of omega-3 fatty acids on the impact of the resolution phase of inflammation should also be emphasized (Furman et al. 2019). Currently, low intake of omega-3 has been reported due to low consumption of fatty fish and high intake of vegetable oils (rich in linoleic acid, which displaces omega-3 fatty acids in cell membrane phospholipids) (Furman et al. 2019; Bachmann et al. 2020).

The healthy effect on immune function was attributed to Mediterranean diet, which is characterized by a high consumption of fruit, vegetables, legumes, nuts, whole grains, fish, and olive oil, whereas moderate intake of dairy products and red wine, and low consumption of red meat (D’Alessandro and Pergola 2018; Morales et al. 2022). Chrysohoou et al. (2004) observed that individuals with higher adherence to the Mediterranean diet had lower levels of CRP, IL-6, homocysteine, fibrinogen, and white blood cell counts compared to those with low adherence to the diet. It follows that Mediterranean diet attenuates inflammatory response and improves vascular endothelial function, what contributes to, among other things, lower cardiovascular and cancer incidences (Sofi et al. 2010; Morales et al. 2022).

Although a properly balanced and varied diet with adequate fluid intake is necessary to maintain health, improves physical performance, and reduces the risk of disease, inadequate consumption of fluids, some vitamins and minerals, and unsaturated fatty acids was observed in firefighters (Raines et al. 2012; Horn et al. 2012; Johnson and Mayer 2020). On the other hand, firefighters consumed the appropriate amount of protein (expressed in grams), not enough carbohydrates, while the consumption of fats was within the upper limits (Johnson and Mayer 2020), which can contribute to the development of overweight and obesity, as well as various chronic diseases. For this reason, healthy eating habits should be implemented, taking into account a well-balanced diet, appropriate to energy expenditure with recommended amounts of vitamins, minerals and other nutrients that could reduce the risk of inflammation and infectious or chronic diseases in this group (Korre et al. 2017; Walsh 2018).

## Strategies of minimizations of inflammation in firefighters

Due to the nature of the work, it is extremely difficult to eliminate the causes of inflammation in firefighters. Therefore, the focus should be on trying to limit the development of inflammatory reaction as much as possible, especially when it is highly dependent on the individual (diet, body weight, stimulants, etc.). To keep firefighters healthy and minimize the potential for inflammation whenever possible, firefighters should consider the following preventive strategies (Table 7).

**Table 7:** Summary of inflammation strategy prevention for firefighters (Walsh 2018; Keaney et al. 2019; Stec et al. 2020)

The cause of inflammation	Practical recommendations
Smoke exposure	• Stay at the fire site as shortly as possible • Limit contact with contaminated areas as much as possible: ○ If possible, rotate firefighters from more contaminated to less contaminated areas ○ If possible, alternate tasks between other firefighters • Wear protective clothing, which fit well, at all times (including during fire and turning over) • Wear face and respiratory protection (breathing apparatus) throughout the entire fire incident, including during salvage and turning over • Remove of hazardous substances after return to the firefighting unit from the body, clothing, equipment and tools to the extent necessary to prevent adverse health consequences: ○ Protective personal equipment should be cleaned and thoroughly decontaminated after each fire incidents in order to avoid the accumulation of toxic contaminants on them ○ When return to the station, after fire exposure, try to take a “shower within an hour” • Protective personal equipment, drills and operational equipment should be kept in “dirty” areas not in the “clean” areas • Try to record and monitor each participation in the fire incidents over the duration of the service • Limit the number of fire exposures by fire instructors
Mental stress	• Kept life stress to a minimum • Monitor the psychological and physical stress and try to manage it • If necessary, stress management interventions should be implemented
Thermal stress	• Wear protective clothing appropriately selected for the working conditions • Wear face and respiratory protection
General health	• Remember about regular medical examinations • Seek medical attention immediately when you notice any disturbing symptoms • Stop smoking and consuming other stimulants
Body mass	• Take care of proper body mass • Reduce body weight when overweight and obese
Work environment	• Maintain good hygiene—it may minimize the risk of infection transmission (wash hand, clean bottles, and equipment) • If you are sick, do not come to work • Avoid self-inoculation (not touching your eyes, nose or mouth)
Sleep disturbance and improper rest	• Follow the rules of sleep hygiene to optimize sleep quantity and quality: ○ Cool and dark bedroom environment (19–22 °C) ○ Avoid stimulants prior to sleep ○ Avoid light-emitting technology devices—TV, phone, computer minimum 30 min before sleep ○ Aim for > 7 h sleep per night at home
Physical activity	• Take care of regular physical activity also outside of work (at least 150 min/week of moderate or 75 min/week of vigorous aerobic activities) to keep high level of physical fitness
Nutrition	• Avoid food with high amounts of salt, fat, sugar or flavor additives • Ensure a varied, well-balanced diet with plenty of fruit and vegetables • Keep yourself hydrated

Detailed information on minimizing contamination during firefighting operations can be found in the “Minimising firefighters’ exposure to toxic fire effluents. Interim Best Practice Report” (Stec et al. 2020).

## Future perspectives

There is still a lack of studies on the effects of occupational exposure, especially smoke exposure on the onset and maintenance of inflammation in firefighters. Therefore, there is a need for continued research in this area in order to obtain conclusive evidence on its impact on firefighters’ health. More human studies are required to estimate the precise effect of harmful combustion products on health. Based on the results of the impact of contaminated air from fires on the human body, an attempt can be made to assess the impact of air pollutants on health also in general population (Swiston et al. 2008). Currently, due to global warming and the increased risk of fires (Smith et al. 2020), understanding the exact impact of toxic combustion products on the immune system, and thus human health, will allow for the development of strategies to reduce the risk of cardiovascular and respiratory diseases and cancer in the future.

## Conclusion

Studies have shown an increased incidence of cardiovascular and respiratory diseases in firefighters, as well as cancer, which can be caused by inflammation induction mainly by exposure to fire. Occupational exposure of firefighters is important for the development of the above-mentioned diseases, but also other factors, such as unbalanced diet, dehydration, obesity, lack of physical activity, mental stress, heat stress or poor sleep should be taken into account when assessing the health burden in this occupational group (Barros et al. 2021). It should be emphasized that it will not be possible to completely eliminate the risk of developing an inflammatory reaction in firefighters, as they will always be exposed to harmful products of combustion, for example. However, attempts can be made to minimize the degree of inflammation by introducing strategies that have anti-inflammatory effects. Education is also very important—firefighters should know what factors cause inflammation and what its effects are. They should also be familiar with methods that can reduce the inflammatory response.

## Data Availability

Not applicable.

## Abbreviations

ACE: Angiotensin-converting enzyme
ACSM: American College of Sports Medicine
AhR: Aryl hydrocarbon receptor
BAL: Bronchoalveolar lavage
BALF: Bronchoalveolar lavage fluid
BMI: Body mass index
CC16: Clara cell protein
CO: Carbon oxide
COVID-19: Coronavirus disease 2019
CRF: Cardiorespiratory fitness
CRP: C-reactive protein
cTnT: Cardiac troponin T
DAMP: Danger-associated molecular patterns
EBC: Exhaled breath condensate
ECP: Eosinophil cationic protein
GM-CSF: Granulocyte-macrophage colony-stimulating factor
Hs-CRP: High sensitivity C-reactive protein
HSP: Heat shock protein
IAFF: International Association of Fire Fighters
IARC: International Agency of Research on Cancer
ICAM: Intercellular adhesion molecule
IgG: Immunoglobulin G
IL: Interleukin
IL-8/ CXCL8: Interleukin 8 or chemokine (C-X-C motif) ligand 8
INF: Interferon
MCP-1/CCL2: Monocyte chemoattractant protein-1, also known as chemokine (C–C motif) ligand 2
MDC: Macrophage-derived chemokine
MET: Metabolic equivalent of task
MIP-1α/CCL3: Macrophage inflammatory protein-1α, also known as chemokine (C–C motif) ligand 3
MMP: Matrix metalloproteinase
NDS-MAC (C): Maximum allowable ceiling concentrations
NDS-MAC (STEL): Maximum allowable short-term concentrations
NDS-MAC (TWA): Maximum allowable concentrations
NLR: Neutrophil-to-lymphocyte ratio
NO_x_: Nitrogen oxides
O_3_: Tropospheric ozone
PAMP: Pathogen-associated molecular patterns
Pb: Lead
PM: Particulate matter
PMN: Neutrophils
PPC: Personal protective clothing
PPE: Personal protective equipment
PRR: Pattern recognition receptors
PTSD: Post-traumatic stress disorder
SAA: Serum amyloid A
SARS-CoV-2: Severe acute respiratory syndrome coronavirus 2
SCBA: Self-contained breathing apparatus
SCD: Sudden cardiac death
sICAM-1: Soluble intercellular adhesion molecule 1
SO_x_: Sulfur oxides
SP-A: Surfactant protein A
sTNFR-1: Soluble tumor necrosis factor receptor 1
TAC: Total antioxidant capacity
TGF: Transforming growth factor
TLR4: Toll-like receptor 4
TNF: Tumor necrosis factor
UFP: Ultrafine particles
VCAM: Vascular cell adhesion molecule
VEGF: Vascular endothelial growth factor
VOCs: Volatile organic compounds
WBC: White blood cells, leucocytes
WHO: World Health Organization
